# Fish-based remedies in Spanish ethnomedicine: a review from a historical perspective

**DOI:** 10.1186/1746-4269-10-37

**Published:** 2014-04-30

**Authors:** José Ramón Vallejo, José Antonio González

**Affiliations:** 1 Departamento de Terapéutica Médico-Quirúrgica, Facultad de Medicina, Universidad de Extremadura, E-06006 Badajoz, Spain; 2 Grupo de Investigación de Recursos Etnobiológicos del Duero-Douro (GRIRED), Facultad de Biología, Universidad de Salamanca, E-37071 Salamanca, Spain

**Keywords:** Ethnozoology, Ethnomedicine, Fish, Medical history, Medical anthropology, Spain

## Abstract

**Background:**

Fish-based therapeutics is fundamentally based on a dietary use, but these vertebrates have also been employed in the treatment of infectious and parasitic diseases, during pregnancy, childbirth and postpartum and to deal with diseases of the different systems.

**Methods:**

An overview of the ethnomedical and historical Spanish literature has been carried out. Automated searches in the most important national and international databases have been performed. All related works have been thorough examined.

**Results:**

We examine the historical use of 54 medicinal fish species, 48 marine and six from inland waters. As useful, in Ancient times 39 species have been recorded (of which only 21 have been collected in subsequent periods), seven in the Middle Ages, 18 in Modern times and 17 in the contemporary period. *Anguilla anguilla*, *Engraulis encrasicolus* or *Scyliorhinus canicula* are species that have survived over time as an ingredient in Spanish folk remedies. Most remedies used in the last century and currently are empirical remedies based on the humorism theory and the principle of *contraria contrariis curantur* (74%), and the rest (26%) are magical type remedies that complete the popular therapeutic arsenal.

**Conclusions:**

In the last century we find a progressive decrease in the number of fish species used in ethnomedicine. Only seven taxa have been documented as surviving therapeutic resources since centuries ago. The existence of a dynamic Spanish ethnomedicine has also been detected which has managed to generate new therapeutic resources in recent times. It is important to validate the remedies by ethnopharmacology and evidence-based medicine. In order to recover as much data as possible, it will be necessary to draw up an inventory of ethnoichthyological uses.

## Background

Ethnozoology is an emerging field in many areas of the world and it is divided, due to its multidisciplinary character [1], into branches of knowledge such as ethnoentomology, ethnoherpetology or ethnoornithology [2-4].

Fishes have a long history of interaction with humans, thus “ethnoichthyology” is acquiring an important role in ethnozoological research [5-7]. There are some studies that discuss the role of ichthyofauna in traditional medicines, mainly in fishing communities [8-12], and that reveal a large number of fish species used in zootherapy, understood as the medicinal use of animals and animal-derived products to treat illnesses and health conditions [13,14].

These works on zootherapy are a very useful tool in the exploration of pharmacologically active substances [15,16]. But also there are other reasons of an anthropological kind for carrying out these ethnozoological studies. For example, they can help us in the understanding of the human behavior toward health care and the use-consumption of fish resources. As well, in many developing countries these studies have a great value in fish diversity conservation [17-19].

In Spain there has not been any ethnozoology development and only very few articles have been published with an ethnobiological approach, although some anthropological, folk and ethnomedicinal studies have focused on the connections between human society and animals [20-23]. This has affected the study of the interactions between humans and fish (ethnoichthyology), and the zootherapy based on these vertebrates is a field of research that has not been given due attention and must therefore be constructed from a framework of an “historical ethnozoology”. Following this philosophy, this paper illustrates the use of fishes in Spanish ethnomedicine and its historical development as a therapeutic resource. It provides an inventory of the species that have been used for medicinal purposes from ancient times to the present, and analyses the medical use of fishes in the 20th century.

Thus, our main objective is to obtain an inventory of the fish species that have been used in Spain for therapeutic purposes from antiquity to the present. From this we determine which medicinal species have survived to recent times and what diseases or medical conditions they have been used for.

## Methods

### Procedures

The present study forms part of a project revision of the ethnozoology in Spain, for which various databases have been examined: ISI Web of Science and Anthropology Plus, JSTOR III - Arts and Sciences, TESEO, the information system of the databases of the Consejo Superior de Investigaciones Científicas (CSIC), the bibliographic website Dialnet, Google Scholar, and the catalogue of Public State Libraries (BPE). The search focused on the type of documents contained in folklore, ethnographic, history of medicine and social and medical anthropology studies. The key words used were: anthropology, history of medicine, ethnomedicine, folk medicine, folklore, zoology, ethnozoology and ethnoichthyology, and the names of the different fish groups in Spanish or English, as appropriate. The search framework also made use of a list of zoonyms contained in Spanish dictionaries [24], and a directory with the names of the most relevant authors of therapeutics throughout history.

The search results were catalogued and the information was classified into ichthyologic groups and historical periods, according to the classical periodization followed by Christoph Cellarius (1638–1707) and those recently employed by De Vos [25]. A total of 52 documents have been analyzed [26-78], of which approximately 8% belong to Ancient history, 10% to Medieval, 10% to “Modern” (6% to 15th-17th centuries and 4% to 18th-19th centuries, respectively), and 72% to “contemporary history”, i.e. from beginning 20th century to present (Table 1).

**Table 1:** List of references consulted

**Ref.**	**Historical period [text used]**
	
	**Ancient**
[26]	Pliny the Elder (23-79 AD) –*Naturalis Historia*– [Cantó *et al*., 2007]
[27]	Pedanius Dioscorides (ca. 49-90 AD) –*De Materia Medica*– [López Eire, 2006]
[28]	Claudius Aelianus (ca. 175-235 AD) [Vara Donado, 1989]
[29]	El libro de San Cipriano (4th century) [facsimile edition 1985]
	
	**Medieval**
[30]	Abd al-Malik Ibn Ḥabib (9th century) [Álvarez de Morales and Girón Irueste, 1992]
[31]	Abulcasis (936-1013) [Arvide Cambra, 2010]
[32]	Ibn Wafid (1008-1074) [Álvarez de Morales, 2006]
[33]	Ibn al-Baytar al-Malaqi (ca. 1180-1248) [Cabo González, 2005]
[34]	Ibn al-Durayhim al-Mawsili (14th century) [Ruiz Bravo-Villasante, 1980]
	
	**“Modern” – 15th-17th centuries**
[35]	Johannes de Cuba –*Hortus Sanitatis* (1491)– [Viñayo and Riesco, 1998]
[36]	Vélez de Arciniega (1613)
[37]	Daza Chacón (1673)
	
	**“Modern” – 18th-19th centuries**
[38]	Palacios (1792)
[39]	Collin de Plancy (1842)
	
	**“Contemporary” – 20th century-at present**
[40]	Nogales (1907)
[41]	Rodríguez López (1910)
[42]	Sánchez Pérez (1948)
[43]	Barriola (1952)
[44]	Cascón (1952)
[45]	Castillo de Lucas (1958)
[46]	Seijo Alonso (1974)
[47]	Alvar (1979-1983)
[48]	Becoña Iglesias (1981)
[49]	Carril (1981)
[50]	Blanco (1985)
[51]	Erkoreka (1988)
[52]	Vázquez Gallego (1989)
[53]	Barandiarán (1990)
[54]	García Arambilet (1990)
[55]	Garmendia Larrañaga (1990)
[56]	Carril (1991)
[57]	Jordán and De la Peña (1992)
[58]	Fragua Gil (1994)
[59]	Gil Barberá and Martí Mora (1997)
[60]	Domínguez Moreno (2000)
[61]	Dueso (2001)
[62]	Erkoreka (2002)
[63]	Álvarez Peña (2004)
[64]	Barandiarán and Manterola (2004)
[65]	Domínguez Moreno (2004)
[66]	González Salgado (2004)
[67]	López Pérez (2004)
[68]	Domínguez Moreno (2005)
[69]	Vallejo *et al*. (2005)
[70]	Hernández Ortega (2007)
[71]	Pérez Vidal (2007)
[72]	Castelló *et al*. (2008)
[73]	Muriel Martín (2008)
[74]	Pardo de Santayana (2008)
[75]	Vallejo (2008)
[76]	Alemany *et al*. (2010)
[77]	Cobo López and Tijera Jiménez (2013)
[78]	Rigat *et al.* (2013)
	

The method of identification of the fish species consisted of a discriminatory analysis of the biological, ecological and ethological information, and of the vernacular nomenclature contained in the works consulted. This was all corroborated with catalogues of names, single-access or dichotomous keys of fish [79-82], and ichthyological databases and species identification websites [83-87]. The assessments have been made from Spanish species, although in some cases taxa from other jurisdictional waters or maritime territories have been considered. Those valid scientific names included in the Species 2000 and ITIS Catalogue of Life: 2013 Annual Checklist [88], are considered in the list of species. The species of most historic importance have been established according to their inclusion in the remedies belonging to the 20th century and at least one previous era.

The illnesses treated with the species mentioned are classified using the chapters of the International Statistical Classification of Diseases and Related Health Problems 10^th^ Revision, ICD-10 (Version: 2010) [89]. Taking into account the species employed in the past century, the relative importance of each group of diseases (IC_i_) in Spanish ethnomedicine has been calculated. A calculation has been made of the relationship between the number of useful species registered for each chapter (C_i_) and the number obtained for the chapter with greatest species richness (C_max_); i.e. IC_i_ = C_i_/C_max_.

### Data analysis

The Spanish ethnomedicine applies to medical ideas and practices with magical, religious and natural (empirical) background [43,45,49,64,71]. In this regard, we have classified all the documented remedies despite difficulties in establishing boundaries for this kind of work review method since remedies based on empirical knowledge are found as well as magical-religious therapies with both empirical and esoteric bases. In accordance with the previously mentioned, the categories for type of remedy are as follows: “magical remedies” and “empirical remedies” (i.e., natural medicine home remedies for those using fish parts for therapeutic means starting from an empirical base).

On the other hand, to evaluate the route of administration of the remedies, we consider two categories: “internal use” and “external use”.

All these are categories make up the variables studied in a test of independence once the values obtained in two-by-two contingency tables were included. The Pearson’s chi-squared test (*χ*^2^) was used to answer the question of no association between variables. This test rejects the null hypothesis (of independence) for statistic values too big to be attributed to random chance. All of this allows us to answer the following key question: The fish-based remedies that have survived in the Spanish ethnomedicine have similar features to those remedies listed in the most relevant historical references?

## Results and discussion

A total of 54 fish species, 48 marine and fresh-water six (*Anguilla anguilla* –a catadromous fish–, *Cyprinus carpio*, *Luciobarbus sclateri*, *Perca fluviatilis*, *Salmo trutta* and *Silurus glanis*), have been identified. In most cases (~90%) the reported vernacular names have been associated with scientific names. Five fish had two or more possible species names associated within the Spanish geographical area. Also, there have been 14 vernacular names that were impossible to match with one taxon, due to a lack of biological, historical and/or philological data; these fishes are named as: “al-manun”, “baraqa”, “cerusa”, “haziba”, “kunckut”, “kurbuy”, “laser”, “lengua de toro”, “pagro de río”, “qita”, “rubayta”, “rubellio”, “sahannah” and “sumayka”. We also found remedies based on the use of “garo/garum”, “fish”, “salted”, “brine fish”, “fish jelly” or “fish tail”. All these data are not included in our study.

Table 2 shows the relationship of inventoried taxa, the vernacular names and the historical times in which their medicinal uses were recorded. A total of 39 fish were used in Ancient times, seven in the Medieval period, 18 in the “Modern” and 17 in the contemporary period. The description of the compiled remedies is included in Table 3.

**Table 2:** Overview of medically important fish species, indicating the different eras in which they have been used

**Fish species**	**Vernacular name(s)**	**Historical periods**			
		**Ancient**	**Medieval**	**Modern**	**Contemporary**
					
*Acipenser sturio* Linnaeus, 1758	Esturión, sollo		✓		✓
*Anguilla anguilla* (Linnaeus, 1758)	Anguila, anguilla	✓	✓	✓	✓
*Argyrosomus regius* (Asso, 1801)	Corvina, curvina, corbina				✓
*Brama brama* (Bonnaterre, 1788)	Japuta		✓		
*Clupea harengus harengus* Linnaeus, 1758	Arenque				✓
*Coris julis* (Linnaeus, 1758)	Julis	✓			
*Crystallogobius linearis* (Düben, 1845)	Gobio	✓		✓	
*Cyprinus carpio* Linnaeus, 1758	Carpa				✓
*Dasyatis pastinaca* (Linnaeus, 1758)	Pastinaca, pastinaca marina	✓		✓	
*Dicologoglossa cuneata* (Moreau, 1881)	Acedía				✓
*Dipturus batis* (Linnaeus, 1758)	Noriega	✓			
*Engraulis encrasicolus* (Linnaeus, 1758)	Anchoa			✓	✓
*Gadus morhua* Linnaeus, 1758	Bacalao				✓
*Galeorhinus galeus* (Linnaeus, 1758)	Galeos	✓			
*Halobatrachus didactylus* (Bloch & Schneider, 1801)	Rata, puerco marino	✓			✓
*Hippocampus hippocampus* (Linnaeus, 1758); *H. guttulatus* Cuvier, 1829	Caballito de mar, caballo marino, echenas, hippocampo	✓		✓	✓
*Lophius piscatorius* Linnaeus, 1758	Rana marina, rana	✓			
*Luciobarbus sclateri* (Günther, 1868)	Barbo				✓
*Merlangius merlangus* (Linnaeus, 1758)	Merlancio, asellus, asselus, borriquito, asno, bacchus	✓		✓	
*Merluccius merluccius* (Linnaeus, 1758)	Merluza				✓
*Mugil cephalus* Linnaeus, 1758	Mújol	✓		✓	
*Mullus barbatus barbatus* Linnaeus, 1758; *M. surmuletus* Linnaeus, 1758	Salmonete, mullus, mulus, mulo	✓		✓	
*Muraena helena* Linnaeus, 1758	Morena, murena	✓		✓	
*Ophisurus serpens* (Linnaeus, 1758)	Ophidion	✓			
*Oreochromis niloticus niloticus* (Linnaeus, 1758)	Coracino	✓			
*Oxynotus centrina* (Linnaeus, 1758)	Porquet, peix porquet, peix porc				✓
*Pagellus erythrinus* (Linnaeus, 1758)	Erythinus	✓			
*Perca fluviatilis* Linnaeus, 1758	Perca	✓		✓	
*Raja clavata* Linnaeus, 1758; *R. microocellata* (Montagu, 1818); *R. miraletus* Linnaeus, 1758; *Leucoraja naevus* (Müller & Henle, 1841)	Raya	✓			
*Remora remora* (Linnaeus, 1758)	Rémora, odynolites	✓			
*Salmo trutta* Linnaeus, 1758	Trucha				✓
*Sarda sarda* (Bloch, 1793)	Sarda	✓			
*Sardina pilchardus* (Walbaum, 1792)	Sardina				✓
*Sciaena umbra* Linnaeus, 1758	Corvallo	✓			
*Scophthalmus maximus* (Linnaeus, 1758)	Rodaballo	✓			
*Scorpaena porcus* Linnaeus, 1758	Escorpión marino, escorpión marino rojo	✓		✓	
*Scorpaena scrofa* Linnaeus, 1758	Cabracho	✓			
*Scyliorhinus canicula* (Linnaeus, 1758)	Lija, perro de mar	✓			✓
*Silurus glanis* (Linnaeus, 1758)	Siluro, ankala	✓	✓	✓	
*Solea solea* (Linnaeus, 1758)	Lenguado	✓		✓	
*Sparisoma cretense* (Linnaeus, 1758)	Escaro	✓			
*Sparus aurata* Linnaeus, 1758	Dorada	✓			
*Spicara maena* (Linnaeus, 1758)	Mena	✓		✓	
*Spicara smaris* (Linnaeus, 1758)	Picarel, smarido, caramel	✓		✓	
*Squalus acanthias* Linnaeus, 1758	Mielga		✓		
*Squatina oculata* Bonaparte, 1840; *S. squatina* (Linnaeus, 1758)	Pez angel, angelote	✓			✓
*Thunnus thynnus* (Linnaeus, 1758)	Atún, cybium (tuna under one year), pelamydes, pelamys (tuna than one year)	✓		✓	
*Torpedo marmorata* Risso, 1810; *Torpedo torpedo* (Linnaeus, 1758)	Tembladera, torpedo, pez torpedo, trimilga	✓		✓	
*Trachinus draco* Linnaeus, 1758	Araña, araña de mar, dragón marino	✓		✓	
*Trachinus radiatus* Cuvier, 1829	Escorpión, escorpión de mar	✓			
*Trachurus trachurus* (Linnaeus, 1758)	Jurel	✓			
*Trigla lyra* Linnaeus, 1758	Trigla, triga	✓	✓		
*Trigloporus lastoviza* (Bonnaterre, 1788)	Rubio, al-santara		✓		
*Uranoscopus scaber* Linnaeus, 1758	Pez rata	✓			

**Table 3:** List of Spanish traditional remedies based on the use of fish

**Fish species**	**Historical period [Ref.]**	**Part(s) used**	**ICD-10**	**Treated ailment**	**Remedy (route of administration–type)**	**Geographical location**
						
*Acipenser sturio*	MED [34]	Meat	I	Rabies	It is useful for rabid dog bite (?)	—
	MED [34]	Meat	XX	Bite and stings from venomous animals	“If a person who has been bitten by a snake eats the crocodile like fish and then drinks wine vomits, this is good and will save them from death. This fish is useful for bites from the horned viper and those of scorpions and salamanders. If the sea hare is crushed in vinegar and applied this also relieves pain” (IN–EMP)	—
	CON [77]	Swim bladder	XIII	Lumbago	Give massages with fish jelly. The higher quality gelatin is obtained from the swim bladder of this species (EX–EMP)	Doñana (Andalusia)
*Anguilla anguilla*	ANC [26]	Whole animal	V	Alcoholism	The drink resulting from drowning two eels in wine … will cause rejection of wine in those who drink it (IN–EMP)	—
	ANC [29]	Blood	V	Drunkenness (inebriation)	To remove drunkenness, it is necessary to prepare a potion with wine and three drops of blood of eel, uttering magic words, in relation to the cabalistic numbers 3, 5 and 7. Once the drunk has taken this concoction, he will be free from drunkenness for the space of one month (IN–EMP)	—
	MED [34]	Bile	V	Mental disorders	Its bile removes madness when it is inhaled (IN–EMP)	—
	MED [34]	—	XI	Liver pain	—	—
	MED [34]	—	XIV	Aphrodisiac	—	—
	MED [34]	—	XVIII	Jaundice (unspecified)	—	—
	MDR [35]	Whole animal	V	Alcoholism	Those who drink wine in which eels have been drowned, develop an aversion to wine (IN–EMP)	—
	MDR [36]	Whole animal, blood	V	Alcoholism	“This property of making wine to be hated, attributed to eel, whenever they are drowned in wine, or if its blood is added to it” (IN–EMP)	—
	MDR [35]	Fat	VIII	Otitis	Its fat cures ears (?)	—
	MDR [36]	Fat	VIII	Diseases of the ears	“Its *enxundia* (fat), says the book entitled The Garden of Health, is used to cure ear diseases” (?)	—
	CON [68]	Meat	IV	Obesity	Obesity is prevented those whose parents ate eels while they were in the womb (IN–MAG)	Talarrubias, Peloche, Herrera del Duque (Badajoz)
	CON [53]	Blood	V	Alcoholism	To cure his vice [of the drunk], we must make him drink the blood of eel, without him knowing. It is thought that drunkenness is remedied by drinking eel blood (IN–EMP)	Basque Country
	CON [64]	Whole animal	V	Alcoholism	There were those who introduced a whole eel into the bottle of wine for them to drink the liquid [without them knowing] (IN–EMP)	Goizueta (Navarra)
	CON [41]	Meat	XIII	Gout	Eel brine ointments (EX–EMP)	Galicia
	CON [52]	Meat	XIII	Gout pain	Brine of eel (EX–EMP)	Galicia
	CON [44,56]	Liver	XV	Facilitate delivery	Eating eel liver is considered optimal (IN–EMP)	Mogarraz (Salamanca)
*Argyrosomus regius*	CON [73]	Otoliths	VII	Eye diseases and their prevention	“Piedra de corvina”… a patient had a stone as a pendant to protect him/her against the diseases of the eye (EX–MAG)	Province of Palencia
	CON [42,71]	Otoliths	XIV	Sterility	“Meagre White Stones”… are considered as a remedy in the irregularities of pregnancy… hang (amulet) around the waist two white stones (EX–MAG)	Canary Islands
	CON [73]	Otoliths	XIV	Nephropathies	“Bone amulet: stone basse” … in our field work we found that this fish amulet has preventive properties and relieve failing “kidney diseases”. We have seen people wear it as a pendant in the neck for nephritic diseases (EX–MAG)	Province of Palencia
	CON [77]	Otoliths	—	Prophylaxis (in general)	This “little stone” is usually carried as a protective amulet. Although formerly many were fishermen that had just in the pocket or in a cloth bag, is now easier to see set in gold or silver as a pendant (EX–MAG)	Doñana (Andalusia)
*Brama brama*	MED [30]	Bile	VII	Eye diseases	Its bile is used in ophthalmic drugs (IN–EMP)	—
	MED [34]	Bile	VII	Eye diseases	Among the types of fish, the *japuta* is balanced. It tends to be hot, because of its speed of movement and how much it jumps. Its bile is more useful for the eyes than that of other fish, and so doctors use it as eye drops. Eye remedies made from bile refresh the body, especially in thin people (IN–EMP)	—
*Clupea harengus harengus*	CON [43]	Meat	I	Worms	Cured herring … which indeed provides an adjuvant action (IN–EMP)	Basque Country
	CON [55]	Meat	II	Sarcoma	A family member of my informant had a leg tumour removed that quickly returned. The diagnosis was sarcoma. In view of this, to cure of this evil it was recommended that he made use of a container used for herrings with several holes in its base. Introducing two live toads into the vessel it was closed with a lid and placed over the sarcoma, tied in position with a bandage. If the toads were still alive the day after having carried out this procedure, then the patient could be expected to recover. However, if the frogs were dead, it meant the irreversibility of the disease. In the house to which I allude, the toads died (EX–MAG)	Basque Country
	CON [54]	Meat	XV	To favour milk production	Eating herrings in abundance (IN–EMP)	Castillejo de Robledo (Soria)
	CON [43]	Whole animal	XVI	For determining the sex of an unborn baby	A superstitious practice consist in put on hot coals a herring, if it jumps or flips it indicates the birth of a boy (EX–MAG)	Basque Country
	CON [51]	Bone	XVI	For determining the sex of an unborn baby	Throwing onto a fire herring bone to see if it jumps or turns (EX–MAG)	Basque Country
*Coris julis*	ANC [26]	Whole animal	XI	Laxative	Broth: releases the bowels. The best soup is made with sea scorpions and rainbow wrasse. It should be cooked with dill, celery, coriander, leek, oil and salt (IN–EMP)	—
*Crystallogobius linearis*	ANC [27]	Whole animal	XI	Laxative	Fresh goby, if you put it into the belly of a pig, sew up well and cook in 12 sextarios [6.5 litres] of water until they are reduced to two, and then let to cool outdoors and drink it, will empty your bowels without discomfort (IN–EMP)	—
	ANC [27]	Whole animal	XX	Dog and snake bites	Applied as a poultice also benefits those bitten (EX–EMP)	—
	MDR [36]	Meat	XI	Relaxing the belly	“He remembers Dioscorides saying: If you put one goby in the stomach of a dog, and sew it up then in twelve parts of water boiled until two, the cooled liquid will, relax the belly without any pain” (IN–EMP)	—
	MDR [36]	Meat	XX	Dog and snake bites	“Applied externally it is also helpful for those who have been wounded by snake, or by dogs” (EX–EMP)	—
*Cyprinus carpio*	CONT [71]	Bile	I	Erysipelas	An excellent remedy for erysipelas is the sweetly anointing with a feather dipped in the bile of carp… putting on top a clean, dry cloth (EX–EMP)	Canary Islands
	CONT [77]	Bone, swim bladder and other leftovers	XIII	Lumbago	Give rubs with jelly fish, obtained from cooking the bones and other debris from these fish (EX–EMP)	Doñana (Andalusia)
*Dasyatis pastinaca*	ANC [26]	Sting	I	Scrofulous tumours	It is good to lance scrofulous tumours, so that there is no wound, with the sting of the stingray, it must be done daily until it is healed completely (EX–EMP)	—
	ANC [26]	Sting	XI	Toothache	Scarifying the gums with the sting sooths them (EX–EMP)	—
	ANC [27]	Sting	XI	Toothache	The sting soothes toothaches, making the teeth fall out (EX–EMP)	—
	ANC [26]	Liver	XII	Skin diseases (lichen, exfoliative dermatitis)	Seal fat removes lichen and exfoliative dermatitis, with three *óbolos* of honey, a stingray liver cooked in oil and the ashes of a sea horse or dolphin applied with water. The treatment, which causes scaring, should be applied after excoriation (EX–EMP)	—
	ANC [26]	Sting	XV	Facilitate delivery	The sting applied to the navel, if taken from a live ray the tossed back into the sea (EX–MAG)	—
	ANC [26]	Whole animal	XX	Antidote	It is its own sting remedy, applied as ash, or another ray with vinegar (EX–EMP)	—
	MDR [36]	Sting	XI	Toothache	“The sting in the tail of the stingray located between the scales pushed up between teeth, mitigates the pain of the teeth, making them fall” (EX–EMP)	—
*Dicologoglossa cuneata*	CON [77]	Meat	XV	As galactagogue	It is suggested the consumption of this fish to improve breast milk production (IN–EMP)	Doñana (Andalusia)
*Dipturus batis*	ANC [26]	Bile	VIII	Ear diseases	Fresh bile is very good for the ears, but also preserved in wine (IN–EMP)	—
*Engraulis encrasicolus*	MDR [35]	Whole animal	I	Scabies	If it is introduced through cut skin, chest scabies is cured with anchovy (IN–EMP)	—
	MDR [35]	—	XX	Bites	It is good against dog bite or sea dragon (?)	—
	CON [70]	Meat	IX	Low blood pressure	Eating salty foods: anchovies (IN–EMP)	La Aparecida (Alicante)
*Gadus morhua*	CON [70]	Meat	I	Tapeworm (infection)	Take cod and lots of water (IN–EMP)	La Aparecida (Alicante)
	CON [50]	Liver	III	Nutritional anaemia	Take cod liver oil (IN–EMP)	Alba de Tormes, Cabeza de Béjar (Salamanca)
	CON [46]	Liver	IV	Loss of appetite (malnutrition)	As a dietary supplement, the most common and popular, … is the cod liver oil (a few tablespoons) (IN–EMP)	Villena (Alicante)
	CON [64]	Liver	IV	Work up an appetite	To return appetite to those who lost it cod liver oil was given (IN–EMP)	Basque Country, Navarra
	CON [69]	Meat	IV	Open appetite	Eaten (IN–EMP)	Guadiana del Caudillo (Badajoz)
	CON [74]	Liver	IV	Lack of appetite	The cod liver oil was taken as a tonic (IN–EMP)	Comarca de Campoo (Cantabria)
	CON [64]	Liver	IV	Rickets	Some locations underscore the importance of taking cod liver oil (IN–EMP)	Basque Country, Navarra
	CON [70]	Liver	IV	Food for strength	Iron and cod liver oil (IN–EMP)	La Aparecida (Alicante)
	CON [75]	Liver	IV	Take strength, restorative	Cod liver oil… taken as food (IN–EMP)	Fuente de Cantos (Badajoz)
	CON [70]	Meat	IX	Low blood pressure (hypotension)	Eating foods high in salt: cod… (IN–EMP)	La Aparecida (Alicante)
	CON [64]	Liver	IX	St. Vitus Dance (Sydenham chorea or chorea minor)	Cod liver oil is given to the sick (IN–EMP)	Abadiano (Biscay)
	CON [78]	Liver	X	Antitussive	Cod liver oil, syrup (IN–EMP)	Eastern Catalan Pyrenees
	CON [76]	Maxillary	XI	Baby toothache	The “gaia” of cod (jawbone), that many children wear around their necks, is gnawed like a dummy to relive teething pain (IN–EMP)	Catalonia
	CON [46]	Maxillary	XI	Encourage good dentition in children	They gave the child a jawbone of cod to bite, “hueso de bacalao” (IN–EMP)	Busot (Alicante); Castillo de Villamafela (Castellón); Adamuz, Aras de Alpuente, Villar del Arzobispo (Valencia)
	CON [59]	Tooth	XI	Encourage good dentition in children	Use a good cod tooth with a hole made in it and hung by a ribbon around the neck, biting that tooth was a good remedy to prevent and ensure good teeth (IN–EMP)	Valencia
	CON [64]	Jaw, fins	XI	Encourage good dentition in children	They gave the child a jawbone of cod to bite, or the round part of the fin in dried cod (IN–EMP)	Basque Country, Navarra
	CON [72]	Gills	XI	Encourage good dentition in children	To relieve the pain of first teeth coming out, a cod gill was given to the child to bite (IN–EMP)	La Vall d’Uixó (Castellón)
	CON [43]	Meat, whole animal	XV	Stimulate the secretion of milk (galactagogue)	To ensure abundant production of milk from the breast, intake of fried cod or cod broth are empirically recommended (IN–EMP)	Basque Country
	CON [49]	Meat	XV	Stimulate the secretion of milk (galactagogue)	Eat salads with cod (IN–EMP)	Comarca de El Rebollar (Salamanca)
	CON [67]	Meat	XV	Stimulate the secretion of milk (galactagogue)	Eat this fish induces liquid intake and produces milk (IN–EMP)	Campo de Cartagena (Murcia)
	CON [43]	Meat	XIX	Seasickness	Habit of chewing salted cod … is usual in our fishermen, at the first sign of imbalance, which even they are not immune (IN–EMP)	Basque Country
	CON [62]	Meat	XIX	Seasickness	Taking a piece of salted cod to be taken, from time to time, a fragment is inserted into the mouth and is consumed slowly (IN–EMP)	Basque Country
	CON [70]	Liver	XIX	Hunger (effects)	Cod liver oil (IN–EMP)	La Aparecida (Alicante)
	CON [64]	Liver	XXI	Convalescence	Given to the sick (IN–EMP)	Abadiano (Biscay)
*Galeorhinus galeus*	ANC [26]	—	XX	Antidote	Cures those stung by stingray(?)	—
*Halobatrachus didactylus*	ANC [26]	—	XX	Antidote	Among the poisonous parts of fish is the *puerco marino* dorsal spine, causing great pain to those affected, the remedy is the “silt” collected from the rest of the body of this fish (?)	—
	CON [48]	Liver	I	Chest infections, when it is expected to be open to having to take all the pus	Put the liver on the top of the boiler of the ship, slowly obtaining a large amount of oil of that liver, called “rat oil". Another way to obtain it is with the same operation but cooking the livers in a pot where it will melt to get rat oil. The oil is used even when healing is well under way (EX–EMP)	Galicia
*Hippocampus hippocampus; H. guttulatus*	ANC [28]	Whole animal	I	Rabies	One roasted them and gave them to the sick so that they are taken and others mashed in vinegar and honey, and thus made into a poultice and applied to the bite wounds, and the result of this operation was that the rabies was dominated in young people, given the desire for water caused in them by the seahorses (IN/EX–EMP)	—
	ANC [26]	Whole animal	V	Urinary incontinence	Roasted and taken with food, they heal urinary incontinence (IN–EMP)	—
	ANC [27]	Whole animal	XII	Alopecia	Whose ashes … once burned, mixed with liquid pine resin or pig lard or marjoram perfume and applied as an ointment, it makes hair sprout on bald patches (EX–EMP)	—
	ANC [26]	Whole animal	XII	Dermatitis	Ash (?)	—
	ANC [26]	Whole animal	XIII	Rib pains	Roasted (IN–EMP)	—
	ANC [26]	Whole animal	XVIII	Fever	Drown seahorses in rose oil to anointing the sick with cold fevers, and placed as amulets on the sick (EX–MAG)	—
	ANC [26]	Whole animal	XX	Antidote	It counteracts the poison of sea hare (?)	—
	MDR [35]	Skin	I	Scabies	Its raw skin is useful for scabies, when mixed with other medicines (EX–EMP)	—
	MDR [35]	Whole animal	XI	Diarrhoea	Burned alive and drunk with sweet wine, cures diarrhoea (IN–EMP)	—
	MDR [35]	Meat	XI	Stomach pains	Dioscorides: The taste of the seahorse is good for the stomach, calms the tummy and is diaphoretic (IN–EMP)	—
	MDR [35]	Whole animal	XII	Alopecia	Seahorse ash burned with all the meat and oil, restores baldness (EX–EMP)	—
	MDR [36]	Whole animal	XII	Alopecia	“As Dioscorides said: A small marine animal, is the Hippocampo, whose ashes mixed with wet pine resin or fat, applied as an ointment, will make hair return to wherever is bald” (EX–EMP)	—
	MDR [35]	Heads	XII	Chloasma (mask of pregnancy)	Crushed and administered with water in the form of ointment, it stops the appearance of patches (EX–EMP)	—
	MDR [35]	Whole animal	XV	Avoid deliveries developed	The seahorse if pregnant women wear them as charms up to the time of delivery, prevents early births, for this it is preserved in salt (EX–MAG)	—
	MDR [35]	Whole animal	XIV	Kidney stones	Crushed with thorns and drunk in wine, they remove kidney stones, sometimes given as solid food (IN–EMP)	—
	MDR [35]	Skin	XIX	Wounds	Burnt, it cleans infected wounds and stops worsening (EX–EMP)	—
	MDR [35]	Whole animal	XIX	Poisoning	This same fish, retains the belly, for which seahorses are very good against *dorycnium* (poison) (IN–EMP)	—
	CON [40]	Whole animal	I	Erysipelas	Amulet: wear a seahorse (hippocampus) as a necklace (EX–MAG)	Province of Badajoz (near Sierra de Aracena)
	CON [47]	Whole animal	XI	Toothache	“They dried seahorses in the pocket because they say toothache cure” (EX–MAG)	Aragon
	CON [66]	Whole animal	XI	Toothache	Carry dried seahorses in your pocket because it is said that they cure toothache (EX–MAG)	Province of Huelva
	CON [45]	Whole animal	XVIII	Headache	—	Levantine area
*Lophius piscatorius*	ANC [26]	Whole animal, heart	I	Dysentery	Marine frogs cooked with sea squill, so as to make pills, or their hearts macerated with honey (IN–EMP)	—
	ANC [26]	Bone	I	Scrofulosis	Pricking is good for scrofulous tumours, so that there is no wound, with a small tail bone of the marine fish called “frog”, it should be done daily until it is completely healed (EX–EMP)	—
	ANC [26]	Whole animal	I	Tetanus	Drinking marine frog broth cooked in oil with salt. For those who have spasms we must add pepper (IN–EMP)	—
	ANC [26]	Whole animal	X	Tonsils (enlargement)	Marine frog broth cooked in vinegar (IN–EMP)	—
	ANC [26]	Whole animal	XII	Skin conditions	Cooked in seawater. It should be cooked until it reaches the consistency of honey (IN–EMP)	—
	ANC [26]	Bile	XII	Malignant ulcers, corrosive and rotten	… worms growing in them are removed with marine frog gall (EX–EMP)	—
	ANC [26]	Whole animal	XIII	Stiff neck	Drinking marine frog broth cooked in oil with salt (IN–EMP)	—
	ANC [26]	Entrails	XIII	Gout and joint diseases	Oil in which marine frog bowels are cooked (EX–EMP)	—
	ANC [26]	Whole animal	XIII	Arthritis	Fresh marine frog calms arthritis attacks, some apply the frogs opened (EX–EMP)	—
	ANC [26]	Fat	XVIII	Headache	Marine frog fat poured drop by drop takes the pain away (EX–EMP)	—
	ANC [26]	Meat	XX	Antidote	Marine frog broth cooked in wine and vinegar is drunk against poisons; … If you eat the meat it is also useful against sea hare and against snakes mentioned above also against scorpions, boiled with wine (IN–EMP)	—
*Luciobarbus sclateri*	CONT [77]	Bone, swim bladder and other leftovers	XIII	Lumbago	Give rubs with jelly fish, obtained from cooking the bones and other debris from these fish (EX–EMP)	Doñana (Andalusia)
*Merlangius merlangus*	ANC [26]	Bile	VIII	Ear diseases	For the ears fresh bile is very good, but also preserved in wine (IN–EMP)	—
	ANC [26]	Otoliths	XIV	Kidney stones	In the head of *bacchus* there are a kind of stone, which swallowed with water are very good for those who have kidney stones (IN–EMP)	—
	ANC [26]	Otoliths	XVIII	Fever	Also the small stones found in the head of the *asellus*, during a full moon is hung in a washcloth as an amulet (EX–MAG)	—
	MDR [38]	Otoliths	XIV	Renal colic, kidney stones	Make a fine powder, and grind down crab eyes, the stones of Perches Fish, and *Merlancio*: Take the blood of Macho, Millet and mealybugs, and add and store for use. They are appropriate for kidney stones, for sands, for nephritic colic, and to excite the urine. Its dosage is half a scruple to a *dragma* (IN–EMP)	—
*Merluccius merluccius*	CON [62]	Liver	IX	Cardiac pathologies	It is food that is considered very good for the heart, and is recommended to those with cardiac pathology… it is usually eaten fried (IN–EMP)	Basque Country
*Mugil cephalus*	ANC [26]	Head	XI	Chafing of the anus	Cured with the ash of mullet heads (EX–EMP)	—
	MDR [35]	Head	XIII	Sciatica	The ash of the head cures (EX–EMP)	—
*Mullus barbatus barbatus; M. surmuletus*	ANC [26]	Whole animal	V	Alcoholism	The resulting drink from drowning a goatfish, … and also from marinated sea grape, causes a rejection of wine in those who drink it (IN–EMP)	—
	ANC [26]	Whole animal, meat	XI	Indigestion	Rubbed or taken in food (IN/EX–EMP)	—
	ANC [26]	Whole animal	XI	Induce vomiting	Preserved, crushed and mixed with drinking (IN–EMP)	—
	ANC [26]	Whole animal, head	XII	Carbuncle	Gets rid of carbuncles rid … the ash of salting goatfish –some use only the head with honey– (EX–EMP)	—
	ANC [26]	Whole animal, meat	XX	Antidote	Against *falangia* (reaper), marine dragons and land scorpions. Rubbed or taken in food (IN/EX–EMP)	—
	ANC [26]	Whole animal, meat	XX	Antidote	It cures those stung by the stingray. Rubbed in or taken in food (IN/EX–EMP)	—
	ANC [26]	Head	XX	Antidote	The head of a fresh goatfish, reduced to ashes, serves against all poisons, especially against those of mushrooms (IN–EMP)	—
	MDR [35]	Whole animal	V	Alcoholism	Those who drink wine in which a goatfish was drowned, will be averse to wine (IN–EMP)	—
	MDR [36]	Whole animal	V	Alcoholism	“The greatest way to make those who get drunk reject wine is to give them the liquid in which a goatfish has been drowned” (IN–EMP)	—
	MDR [36]	Whole animal	V	Alcoholism	“And causes tedium of wine to drink that wine in which they were drowned” (IN–EMP)	—
	MDR [35]	Whole animal	XII	Carbuncle	The ash of salted goatfish (EX–EMP)	—
	MDR [36]	Whole animal (salting)	XII	Carbuncle	Goatfish ash salt … salt with which they have been salted, cures carbuncles (EX–EMP)	—
	MDR [35]	Head	XIII	Sciatica	The ash of several goatfish heads, cures sciatica sufferers: burn in a clay pot, and use as an ointment mixed with honey (EX–EMP)	—
	MDR [35]	Meat	XIV	Menstruation problems	Helps women when menstruating (IN–EMP)	—
	MDR [35]	Head	XIX	Mushroom poisoning (toxic effect)	The fresh head ash is good against any type of poison, and particularly against fungi (IN–EMP)	—
	MDR [36]	Head	XIX	Mushroom poisoning (toxic effect)	The ash from the head against all poisons, and primarily against fungi (IN–EMP)	—
	MDR [35]	Whole animal, meat	XX	Poisonous stings	Pliny: goatfish is good to make an ointment against stingrays, both terrestrial and sea scorpions (rockfish), dragons and *falangia*; also good if eaten (IN/EX–EMP)	—
	MDR [36]	Whole animal	XX	Poisonous stings	“In the book entitled *Hortus Sanitatis*, Pliny says that: The eating of *mulo* fish or rubbed in, can aid against the damage caused by stingrays, terrestrial and marine scorpions, marine dragons and *falangios*” (IN/EX–EMP)	—
	MDR [36]	Whole animal, meat	XX	Antidote	“Dioscorides says about these fish: *mulo* being eaten very frequently seems to obscure the sight. Uncooked and cut into pieces, and applied as a poultice, it cures the bites of marine dragons, and of scorpions and spiders” (EX–EMP)	—
*Muraena helena*	ANC [26]	Head	XX	Bite	Bites from moray are cured by the ash of the head of the same fish (EX–EMP)	—
	MDR [35]	Whole animal	I	Leprosy	Moray ash, with three *óbolos* of honey (EX–EMP)	—
	MDR [35]	Whole animal	XII	Lichen	Moray ash, with three *óbolos* of honey (EX–EMP)	—
*Ophisurus serpens*	ANC [26]	Whole animal	XIV	Urinary incontinence	*Ophidion* … a little fish similar to the conger, with iris root, and the small fish removed that it has eaten, burned, and the ashes taken with water (IN–EMP)	—
*Oreochromis niloticus niloticus*	ANC [26]	Meat	I	Abscesses	The salt of salted *coracinos* dissolves abscesses (EX–EMP)	—
	ANC [26]	Bile	VII	Sharpen sight	The bile of *coracino* also sharpens of sight (IN–EMP)	—
	ANC [26]	Meat	XII	Carbuncle	An ointment of salted *coracinos* reduces carbuncles (EX–EMP)	—
	ANC [26]	Meat	XX	Antidote	Its meat … with a topical application is effective against scorpions (EX–EMP)	—
*Oxynotus centrina*	CON [76]	Liver	XIX	Heal burns	Liver oil of the “peix porquet” (EX–EMP)	Costa Brava (Gerona)
	CON [76]	Liver	XX	To relieve pain in case of poisonous fish bite (fish spider, scorpion fish)	Fishermen produce oil with fish liver. Heat the oil and apply it with a cloth (EX–EMP)	Costa Brava (Gerona)
*Pagellus erythrinus*	ANC [26]	Meat	XI	Laxative	If eaten *erythini* act as a laxative (IN–EMP)	—
	ANC [26]	Meat	XIV	Increases libido	Eat (IN–EMP)	—
*Perca fluviatilis*	ANC [26]	Vertebra	I	Quartan fevers (malaria)	They say that carrying a perch vertebra as a charm cures fever (EX–MAG)	—
	ANC [26]	Head	I	Abscesses	Ash of head in brine, adding honey (EX–EMP)	—
	ANC [26]	Head	II	Carcinomas	Heads in brine stop carcinomas, more effective when the ashes are mixed with salt and *Satureja* and made into a paste with oil (EX–EMP)	—
	ANC [26]	Head	XV	Problems uterus, expulsion of afterbirth	The ash of perch heads with salt, savory and oil cures the matrix, and in fumigation expels the afterbirth (EX–EMP)	—
	MDR [38]	Otoliths	XIV	Renal colic (kidney stones)	Make a fine powder, and grind down crab eyes, the stones of Perches Fish, and *Merlancio*: Take the blood of Macho, Millet and mealybugs, and add and store for use. They are appropriate for kidney stones, for sands, for nephritic colic, and to excite the urine. Its dosage is half a scruple to a *dragma* (IN–EMP)	—
*Raja clavata*; *R. microocellata*; *R. miraletus*; *Leucoraja naevus*	ANC [26]	Liver	XII	Pruritus, eczema	The liver of ray cooked in oil very effectively calms them (EX–EMP)	—
*Remora remora*	ANC [26]	Whole animal	XIV	Inhibit sex drive	Applied to the genitals inhibits the loving impulse (EX–EMP)	—
	ANC [26]	Whole animal	XV	Nesting, early delivery	Hung around the neck, they delay pregnancies with a premature tendency, and others, if kept in salt and used as an amulet, precipitates labour, and therefore are called by a different name, *odynolites* (EX–MAG)	—
*Salmo trutta*	CON [49]	Tail	V	Encourage early and proper development of language	For the correct development of language introduce into the infant’s mouth a trout tail (IN–EMP)	Sierra de Francia (Salamanca)
*Sarda sarda*	ANC [26]		XX	Antidote	Against *prester* (viper) bite (IN–EMP)	—
*Sardina pilchardus*	CON [57]	Heads, innards	XII	Sore feet and blisters	Rub sore and blistered feet with the intestines and heads of salty sardines (EX–EMP)	Sierra de Segura (Albacete)
	CON [42]	Whole animal	XVI	Forecast sex	Put a sardine to the fire, in the presence of the pregnant woman. Failure to release the scales, will mean a male, and if the scale jumps then a female. In Huesca the reverse is said (EX–MAG)	Burgos, Huesca
	CON [43]	Bone	XVI	For determining the sex of an unborn baby	Another type of superstitious practice … put on hot coals the bone of a sardine, if it jumps or flips it indicates the birth of a boy (EX–MAG)	Basque Country
	CON [51]	Bone	XVI	For determining the sex of an unborn baby	Throwing a sardine bone onto the fire to see if it jumps or flips (EX–MAG)	Basque Country
	CON [61]	Bone	XVI	For determining the sex of an unborn baby	Cast on to the grill sardine bones. It was said that “if it turns, there will be son if not, a daughter”. In … [other places] it was said “if it jumps a boy if it falls a girl” (EX–MAG)	Larraun, Lekaroz, Irurita (Navarra)
	CON [46]	Whole animal	XVIII	Bloating	A cure based on the application on the stomach of a poultice made by kneading sardines in a mortar with chopped celery, adding barley flour and sprinkling it with vinegar (EX–EMP)	Villena (Alicante)
	CON [46]	Whole animal	XVIII	Fever (unspecified)	Apply to sole of the foot a salty sardine half open, and then bandaging (EX–MAG)	Benilloba (Alicante); Villores, Castillo de Villamalefa (Castellón)
	CON [63]	Head	XIX	Chilblains	When were stale, sardine heads were baked and the resulting water was used to wash chilblains (EX–EMP)	Pandón de Lada –Langreo– (Asturias)
	CON [70]	Head	XIX	Chilblains	Giving rubs with the head of a sardine (EX–EMP)	La Aparecida (Alicante)
	CON [46]	Innards	XIX	Chapped hands	Cracks are rubbed with innards of salted sardines (EX–EMP)	Comarca de Los Serranos (Valencia)
*Sciaena umbra*	ANC [26]	Intestines, scales	XII	Abscesses	It dissolves them (EX–EMP)	—
*Scophthalmus maximus*	ANC [26]	Whole animal	III	Diseases of spleen	It is good to apply them, then throw into the sea (EX–MAG)	—
*Scorpaena porcus*	ANC [26]	Bile	I	Warts	Removes warts (EX–EMP)	—
	ANC [26]	Bile	VII	Incipient cataracts, eye white spots	Bile with rancid oil or Attic honey. Rub on eyes three times leaving several days in between applications (IN–EMP)	—
	MDR [36]	Bile	VII	Cataracts, clouds and vision problems	Talking about this fish Dioscorides, says: The bile of marine scorpion, is helpful against cataracts, against clouds, and other weaknesses of sight (IN–EMP)	—
*Scorpaena scrofa*	ANC [27]	Bile	VII	Diseases of the eye	The bile of red scorpionfish is good for cataracts, walleye, amblyopia (IN–EMP)	—
*Scyliorhinus canicula*	ANC [26]	Tooth	V	Sudden terrors	As an amulet (EX/MAG)	—
	ANC [26]	Encephalon	XI	Toothaches	It sooths by scarifying the gums (IN–EMP)	—
	CON [62]	Skin	V	Behaviour modification in children who cleaned the snot with the forearm	… To these children a piece of fish skin was placed in the sleeve, thus making them abandon this bad habit (EX–EMP)	Basque Country
*Silurus glanis*	ANC [27]	Whole animal	I	Dysentery	Its brine, in sitting baths, is good to those who begin to suffer from dysentery, because it attracts out flows and as enema (IN/EX–EMP)	—
	ANC [26]	Head	I	Erysipelas	Salted wels catfish head ash dissolved in vinegar (IN–EMP)	—
	ANC [26]	Liver	I	Warts	Used topically (EX–EMP)	—
	ANC [26]	Head	VII	Serpiginous ulcers	The ash of the head of catfish stops serpiginous ulcers, and their excretions (EX–EMP)	—
	ANC [26]	Meat	X	Improves voice	Fresh or salted (IN–EMP)	—
	ANC [27]	Meat	X	Tracheitis, voice	Salted wels catfish … is not nutritious but it purifies the trachea and tempers the voice (IN–EMP)	—
	ANC [27]	Meat	XI	Belly troubles	Fresh wels catfish, eaten, is nutritious and beneficial for the stomach (IN–EMP)	—
	ANC [26]	Meat	XI	Laxative	Wels catfish in its juices releases the belly (IN–EMP)	—
	ANC [26]	Whole animal	XII	Pyoderma gangrenosum	Phagedenic ulcer is cured with rancid catfish crushed with *rejalgar* (EX–EMP)	—
	ANC [26]	Meat	XIII	Sciatica	The brine of wels catfish, administered as enema, after evacuation (IN–EMP)	—
	ANC [27]	Whole animal	XIII	Sciatica	And its brine, in a sitting bath, …, cures those affected by sciatica (EX–EMP)	—
	ANC [26]	—	XV	Facilitate delivery	It is also said that fumigation of catfish, especially if it is African make simpler deliveries (EX–EMP)	—
	ANC [26]	Meat	XIX	Foreign bodies in the skin	Splinters lodged in the body are extracted with … the use of river catfish meat (EX–EMP)	—
	ANC [27]	Meat	XIX	Expel thorns	Salted meat, applied as a poultice, expels splinters (EX–EMP)	—
	ANC [26]	Fat	XIX	Chilblains	Cured by means application of wels catfish fat (EX–EMP)	—
	MED [34]	Meat	IV	Metabolism	When you eat the flesh of this fish, if eaten fresh, it nourishes and slows nature. If salted less so (IN–EMP)	—
	MED [34]	—	X	Apnoea, throat	It’s useful for shallow breathing and useful for a good voice (?)	—
	MED [34]	Whole animal	XI	Intestinal ulcers	The salting water is good for intestinal ulcers if you make a enema with it, also if the person sits on top (IN–EMP)	—
	MED [34]	—	XII	Dandruff, oily hair	If placed as ashes on the head, it removes grease and dandruff (EX–EMP)	—
	MED [34]	Bile	XIII	Sciatica	The bile, mixed with musk, is good for sciatica (EX–EMP)	—
	MDR [36]	Meat	I	Dysentery	“If you bathe in its brine, it is helpful at the beginning of the dysentery, because it attracts to the surface the humours that are in the belly” (EX–MAG)	—
	MDR [36]	Meat	X	Bronchitis, clear your throat	“it also clears the tubes of the lungs and clears the throat” (IN–EMP)	—
	MDR [36]	Meat	XI	Soften the belly	“According to Dioscorides, speaking about its properties says: Freshly cooked Catfish, nourishes and softens the belly, but salted offers very little maintenance” (IN–EMP)	—
	MDR [36]	Meat	XIII	Sciatica	“It heals sciatica, taken after fasting” (IN–EMP)	—
	MDR [36]	Meat	XX	Chips kneeling	“It removes swollen splinters from any part of the body” (EX–EMP)	—
*Solea solea*	ANC [26]	Whole animal	II	Splenic tumour (malignant neoplasm of spleen)	It is good to apply, then throw it into the sea (EX–MAG)	—
	MDR [35]	Meat	IV	Sick diet	According to doctors, it is a very light food for the sick, it is less phlegmatic than others … (IN–EMP)	
*Sparisoma cretense*	ANC [28]	Bile	XVIII	Jaundice	If a sick man you give him *escaro* bile to eat then he will heal, as taught by experienced fishermen (IN–EMP)	—
*Sparus aurata*	ANC [26]	Meat	XX	Antidote	Where there is poisonous honey produced, the remedy is to eat gilt-head bream (IN–EMP)	—
*Spicara maena*	ANC [26]	Head, meat	I	Abscesses	Ashes of *mena* head, and the cooked meat are applied (IN/EX–EMP)	—
	ANC [26]	Head	I	Warts	The ash of *mena* head crushed with garlic (EX–EMP)	—
	ANC [26]	Head	I	Genital warts	Ashes of the head (EX–EMP)	—
	ANC [26]	Head	VII	Serpiginous ulcers	The ash of *mena* head stops serpiginous ulcers, and their excretions (EX–EMP)	—
	ANC [26]	Head	X	Tonsils	Ash of salted heads applied with honey (EX–EMP)	—
	ANC [26]	Head	XI	Anal fissures	Ashes of the head (EX–EMP)	—
	ANC [27]	Head	XI	Anal fissures	The burnt head, pulverized, removes anal cracks (EX–EMP)	—
	ANC [27]	Guts	XI	Mouth sores	The “garo” (*garum*) used as a mouthwash heals festering sores in the mouth (IN–EMP)	—
	ANC [26]	Whole animal, head	XI	Mouth ulcers	The brine from the *mena* and ash of the head heal mouth ulcers (IN–EMP)	—
	ANC [26]	Whole animal	XI	Laxative	Salted *mena* spread on the naval with bull bile causes the release of the belly (EX–EMP)	—
	ANC [26]	Head	XII	Nail harshness	The ash of *mena* heads soften the rough edges of nails (EX–EMP)	—
	ANC [26]	Head	XV	Problems uterus, expulsion of afterbirth	The ash of the heads of *mena* heads with salt, savory, and oil cure the matrix, and as a fumigation expel the afterbirth (IN–EMP)	—
	MDR [36]	Whole animal	XI	Mouth sores	“Its brine dries up mouth sores full of corruption, if used as mouthwash” (IN–EMP)	—
	MDR [36]	Head	XI	Anal fissures	“About this fish Dioscorides says: The ash from head of salted *Menas*, heals the anal cracks” (EX–EMP)	—
*Spicara smaris*	ANC [26]	Whole animal	I	Warts	Crushed and applied on warts (EX–EMP)	—
	ANC [27]	Head	I	Scab and plantar warts	The burnt head of salted *caramel* reduces scab and warts … (EX–EMP)	—
	ANC [27]	Head	XIX	Healing wounds and ulcers	The salted burnt head of the *caramel* reduces sores and stops fleshy excretion and infection (EX–EMP)	—
	ANC [27]	Head	XX	Bites and stings	The salted burnt head of the *caramel*… goes well against scorpion stings and dog bites (EX–EMP)	—
	ANC [26]	Meat	XV	Increase milk production	Taken with barley or cooked with fennel (IN–EMP)	—
	MDR [36]	Head	I	Scab and plantar warts	Removes hardened scab and warts (?)	—
	MDR [36]	Head	XII	Ulcers	“The *Smarido* head, salted and burnt, represses the growth on the wounds and reduces those about to break out” (EX–EMP)	—
	MDR [36]	Meat	XX	Dog bites	“Its salted meat, like almost all brine, is helpful against dog bites” (EX–EMP)	—
	MDR [36]	Meat	XX	Scorpion stings	“Its salted meat, like almost all brine, is helpful against scorpion stings” (EX–EMP)	—
*Squalus acanthias*	MED [32]	Whole animal	VI	Convulsions	Crush, cook and make a poultice (EX–EMP)	—
*Squatina oculata; Squatina squatina*	ANC [26]	Skin	I	Abscesses	Applying the burnt skin (EX–EMP)	—
	ANC [26]	Whole animal	XIV	Stop breast growth	The application of angelshark impedes the growth of the breasts (EX–MAG)	—
	CON [66]	Roe	X	Asthma	“Angelote” (*Squatina squatina* L.) … its roe (eggs) collected on Good Friday cure asthma (IN–MAG)	Gran Canaria (Canary Islands)
*Thunnus thynnus*	ANC [26]	Head	I	Abscesses	Applying head ash (EX–EMP)	—
	ANC [26]	Head	I	Genital warts	The head of salted *pelamys* [one year old tuna] with honey (EX–EMP)	—
	ANC [26]	Meat	XI	Toothaches	Rancid tuna, washed in a bowl and crushed is effective (IN–EMP)	—
	ANC [26]	Head	XI	Anal fissures	The head of salted *pelamys* [one year old tuna] with honey (EX–EMP)	—
	ANC [26]	Meat	XII	Malignant ulcers, corrosive and rotten	With pieces of stale tuna (EX–EMP)	—
	ANC [28]	Blood	XII	Depilatory	If a young man desires to be without beard for a very long time, if he smears that part with tuna blood he will remain hairless (EX–EMP)	—
	ANC [27]	Whole animal	XX	Dog bite	Apply as a poultice (EX–EMP)	—
	ANC [27]	Meat	XX	Viper bite	The so-called “salting raw” (*ōmotárikhos*) is preserved salted tuna meat. If taken it assists those bitten by the snake called “that which swells” (*prēstêr*), it is necessary to add the maximum amount of wine and to drink heavily to force them to vomit (IN–EMP)	—
	ANC [26]	Meat	XX	Antidote	Against the sea dragon (IN–EMP)	—
	MDR [36]	Meat	XI	Indigestion	“It’s also useful to provoke the vomiting of meals that bother the stomach” (IN–EMP)	—
	MDR [35]	Liver	XII	Cosmetics	Its liver, chopped and mixed with cedar oil, preserved in a lead chest, outline the eyelids (EX–EMP)	—
	MDR [35]	Blood, bile, liver	XII	Depilatory	From the blood, bile and liver of tuna, fresh or preserved, a cream is made (EX–EMP)	—
	MDR [35]	Whole animal	XX	Dog bites	Applied in poultices (EX–EMP)	—
	MDR [36]	Meat	XX	Dog bites	Useful applied against dog bites (EX–EMP)	—
	MDR [35]	Meat	XX	Snake bites (antidote)	Dioscorides: If you eat a lot of salted tuna, harm from snakebite is reduced (IN–EMP)	—
	MDR [36]	Meat	XX	Viper bites	“Salted tuna, which is called in the Greek language *Omotarichos*, if eaten in large quantities, cures the bites from the viper called *Prester*. But you should give it with a large amount of wine, and force them to vomit” (IN–EMP)	—
*Torpedo marmorata; Torpedo torpedo*	ANC [26]	Whole animal	III	Spleen diseases	It is good to apply, then throw it into the sea (EX–MAG)	—
	ANC [26]	Whole animal	XI	Laxative	Marbled electric ray in its juices releases the belly (IN–EMP)	—
	ANC [27]	Whole animal	XI	Rectal prolapse	Applied in the anus it reduces anal prolapse (EX–EMP)	—
	ANC [28]	Meat	XII	Depilatory	If its meat is subjected to a process of putrefaction in vinegar and smeared onto the chin, the effect is said to be the disappearance of hair (EX–EMP)	—
	ANC [26]	Bile	XIV	Inhibits sex drive	Applied to the genitals it inhibits the sexual impulse (EX–EMP)	—
	ANC [26]	Whole animal	XV	Facilitate delivery	If you capture a marbled electric ray when the moon is in the sign of Libra and keep it for three days outdoors, then deliveries are made easy, as often as it is presented to a woman in labour (EX–MAG)	—
	ANC [27]	Whole animal	XVIII	Headache	Applied in cases of chronic headaches, it alleviates the severity of the pain (EX–EMP)	—
	MDR [35]	Whole animal	III	Spleen diseases	Used as a poultice, it cures the spleen (EX–EMP)	—
	MDR [35]	Meat	XI	Digestive	Pliny, book 32: Taken as food, the marbled electric ray softens the belly (IN–EMP)	—
	MDR [35]	Whole animal	XI	Hernia	Placed on top it inhibits the illness that causes the fall of the intestine (EX–EMP)	—
	MDR [36]	Meat	XVIII	Headache	“…because as Galen says, it has the virtue of removing headache, placed on it, and also so says Dioscorides, that it relaxes the head” (EX–EMP)	—
*Trachinus draco*	ANC [26]	Bones	XI	Toothache	Sea dragon bones soothe the gums (IN–EMP)	—
	ANC [26]	Meat	XX	Stings	The sea dragon sting is cured by directly applying its own meat (EX–EMP)	—
	ANC [26]	Whole animal, brain	XX	Antidote	Against the venom of the sting of the sea dragon the application of the fish itself is effective, or a potion made from its own brain (EX–EMP)	—
	ANC [26]	Meat	XX	Antidote	The sting is cured by applying the meat (directly) (EX–EMP)	—
	ANC [27]	Whole animal	XX	Antidote	… opened up and applied over the wound is a cure for its own sting (EX–EMP)	—
	MDR [36]	Whole animal	XX	Wound he did with his own spine	“Dioscorides says: the Marine Dragon, opened and applied, heals the wound caused by its own sting” (EX–EMP)	—
	MDR [35]	Whole animal	XX	Antidote	Pliny… Likewise, the sea dragon itself is good if it placed as a poultice against the venom of its sting, which caused the wound./Avicenna… The same author in the second book: As Galen says, the sea dragon is sliced and placed on their own bite as a cure (EX–EMP)	—
*Trachinus radiatus*	ANC [26]	Whole animal	I	Abscesses	Cooked in wine, thereby making poultices, the abscesses are dissolved (EX–EMP)	—
	ANC [26]	Whole animal	XI	Liver pain	Drowned in wine, then drank (IN–EMP)	—
	ANC [26]	Bile	XII	Scars	Applied directly removes scars (EX–EMP)	—
	ANC [26]	Meat	XX	Antidote	The sting is cured by applying the meat (EX–EMP)	—
*Trachurus trachurus*	ANC [26]	Liver	I	Mumps	—	—
*Trigla lyra*	ANC [27]	Meat	XX	Bites, antidote	The *trigla*, raw, split and applied it cures the bites of sea spider, sea scorpion and spiders (EX–EMP)	—
	MED [34]	Skinned animal	XII	Pustules	The *triga* skinned and placed on the pustules of the body, cures and heals them (EX–EMP)	—
	MED [34]	Whole animal	XX	Foreign bodies deep in the body	Crushed and smeared on a site where there is an arrowhead or a thorn stuck deep in the body, it extracts them thanks to its vigorous nature and amazing properties (EX–EMP)	—
*Trigloporus lastoviza*	MED [34]	Whole animal	XX	Contact with venomous animals	If cut up and placed on a jellyfish, scorpion or spider sting, it feels good (EX–EMP)	—
*Uranoscopus scaber*	ANC [26]	Bile	VII	Eye wounds	Heals scars and removes growths from the eye. No other fish has such an abundant bile (IN–EMP)	—

Historical periods: ANC = Ancient; MED = Medieval; MDR = “Modern” (15th-19th centuries); CON = “Contemporary” (20th century-at present). Route of administration: IN = internal use; EX = external use. Type of remedy: MAG = magical remedy, EMP = empirical remedy.

### On the determination of the species

There is no doubt that the identification of species that appear in historical and classical works is inherently difficult. In relation to this problem on taxonomic interpretation Josefa Cantó writes:

“The difficulties are varied: scarcity and inconsistency of data-often saying incompatible things about the same species in different-passages; even Pliny makes mistakes, sometimes he misinterprets his sources and combines names and characteristics that do not correspond to the same species. It contributes to the confusion that sometimes he uses two different names, one Greek and one Latin, for the same fish. Besides data from ancient texts about size, colour, habitat, habits, etc., the etymology of the names or their possible survival in Romance languages may help. It’s important to enlist the help of biologists since their knowledge of marine species allows a better guess at what may lay hidden behind a superficial description” [26].

We include some fragments of the work by Francisco Vélez de Arciniega published in 1613, entitled *Historia de los animales mas recebidos en el uso de medicina: donde se trata para lo que cada uno entero ò parte del aprovecha y de la manera de su preparacion*…, which has been useful to make a differential diagnosis of some species. This work is of special interest because it synthesizes descriptions of Aristotle, Dioscorides, Pliny and Galen, among other classic authors.

In the fourth book *De los pescados recebidos en el uso de la Medicina*, Vélez de Arciniega “De las Menas” and “Del Smarido” tells us that:

“Menas are very similar to Smaridos, which (according to Pliny) change colour at different times of the year, and are white in the winter and black in the summer. They swim collectively in the sea, near the shore” [36].

The differential diagnosis leads to *Spicara maena* and *S. smaris*. The “mena” is *S. maena*, which presents a very variable coloration according to sex, age and season. *Spicara smaris*, the “smarido”, unlike which has a silvery, gray back and as the text indicates swims in important shoals.

“Del Gobio” we get *Crystallogobius linearis*:

“It is a small fish the gobio, which has a big mouth in proportion to the body: belly it has many strands close to its belly, it is glutinous: and so very easily be slips between the hands. According to Aelianus: The Gobio, Dragon and Sea Swallow, pitch poison, but it is not fatal … They lay (according to Aristotle) their eggs on the banks, against the stones and in the sands” [36].

This description is compatible with *C. linearis*, which has a size of 4.7 cm in the male and 3.9 cm in the female, it has a big mouth, its first dorsal fin has two conspicuous radios in the male and external muscle segments just as suggested the text. Its range includes the Atlantic (it goes as far as Gibraltar) and the Mediterranean. It is a benthic species commonly found on shells and on sandy bottoms. In Dioscorides, some authors associate the word “gobio” to the *Gobio gobio* species [27], which is a freshwater fish with a small mouth and which lives in sediment covered beds. Andrés de Laguna, commenting Dioscorides, states that:

“The gobio is a well known fish on all the shores of the Adriatic Sea, although it is also found in some lakes and rivers, the head of the gobio is in proportion to the body, very great: it is very tasty and easily digested, having very delicate meat and is a good nutrient, mainly those which are caught between some rocks” [90].

From the explanation in “Del Scorpion Marino” we get *Scorpaena porcus*:

“The Scorpion grows within the Pielago, and Scorpina in more swampy places. Scorpion is red in colour and Scorpina almost black. “He” is good gentle food and “she” not so. Scorpion has a flap on the back with twelve sharp spikes, and a very large spiked head: it has short, thick teeth and very small, thin almost invisible scales” [36].

The morphological character, a large head and a dorsal fin with 12 hard spines and 10 soft rays allows the identification of *S. porcus*. These characteristics of the species distinguished it from *Trachinus radiatus* also known in Spain as “escorpión” (scorpion) or “araña” (spider), which is of a dark colour on the back and has six hard poisonous rays in the first of the two dorsal fins. It should be noted that Pliny refers to both the dragon and the scorpion when he writes “which have stings in the gills facing the tail, and thus sting like a scorpion when you pick them up” [26]. In this case the “scorpion” would be *T. radiatus*, in addition to the characteristics described, presents poisonous opercula spines oriented toward the tail.

### Ethnomedical practices: useful species and remedy features

**Figure 1:**
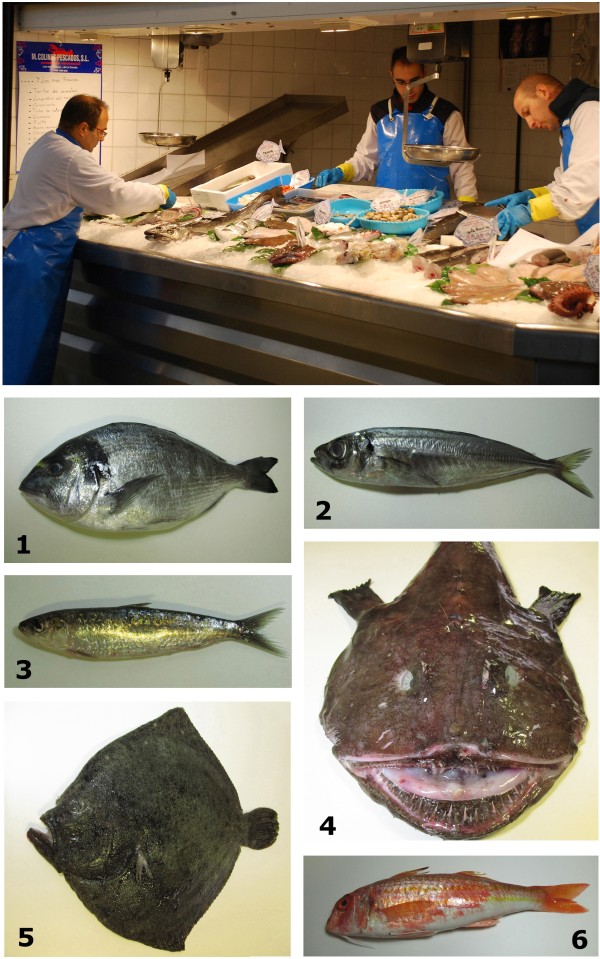
**Examples of medicinal fishes traded in Spanish fish markets and consumed as food.** (1) gilt-head bream, *Sparus aurata*; (2) Atlantic horse mackerel, *Trachurus trachurus*; (3) sardine or European pilchard, *Sardina pilchardus*; (4) angler, fishing-frog, frog-fish or sea-devil, *Lophius piscatorius*; (5) turbot, *Scophthalmus maximus*; (6) striped red mullet, *Mullus surmuletus*.

Many species documented in this study as useful in the therapeutic are species currently marketed and consumed in Spain (Figure 1). The most historically important fish species, with use-reports in the 20th century and in one or more past periods, and whose usage has survived over time, are: *Acipenser sturio*, *Anguilla anguilla*, *Engraulis encrasicolus*, *Halobatrachus didactylus*, *Hippocampus* sp., *Scyliorhinus canicula* and *Squatina* sp. (see Table 2).

**Figure 2:**
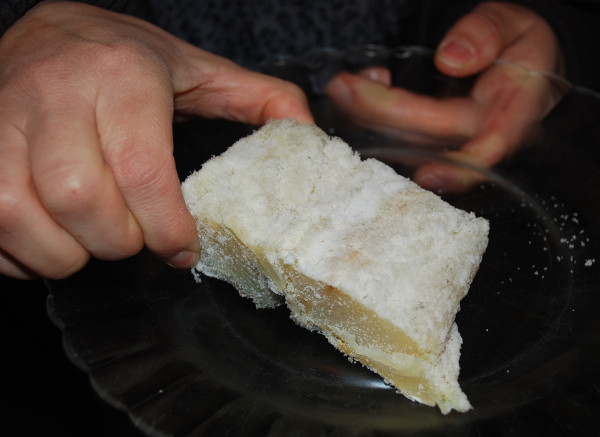
**A bit of dried and salted cod.** In Spain the “bacalao” is used in the culinary preparation of numerous and diverse traditional dishes. This product is consumed raw in salads, stewed or eaten fried.

Cod (*Gadus morhua*) is the species for which we have found a large number of use-reports (24). Although some remedies are based on the use of its jawbones, fins or gills, the importance of this species lies in the frequent use of cod liver oil to treat nutritional problems and the intake of meat of dried and salted cod to stimulate the secretion of milk and prevent seasickness. At this point, it should be noted that the salt cod was for many decades the only fish stored and consumed by the rural communities of the central part of Spanish territory (Figure 2).

**Figure 3:**
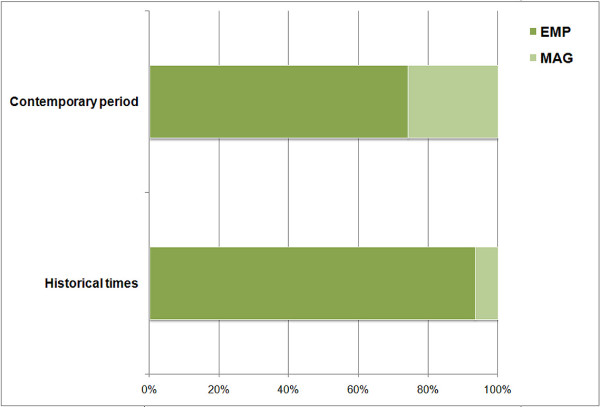
**Relative importance of the two remedy types considered throughout Spanish medical history.** EMP = empirical remedies, MAG = magical remedies.

According to the results obtained from the statistical analysis performed to evaluate the features of the different remedies found, we can conclude that the route of administration of a remedy and the periodization (historical periods *vs.* contemporary times) are independent (*χ*^2^ = 2.850, df = 1, p = 0.091). By contrast, there does exist an association between the type of remedy and the periodization (*χ*^2^ = 15.751, df = 1, p < 0.0001), they are not independent. The number of fish-based empirical remedies is significantly larger as well as the number of magical remedies in the case of the historical periods (Figure 3). These differences are not due to random chance.

### Concerning therapeutic foundations and medicinal value

Magic and superstition are poorly understood concepts in some contexts and situations. Many remedies can be labelled as superstitions or magic, but is all that is branded superstitious or magical really so? From an anthropological viewpoint magic can be defined as “a combination of beliefs and practices based on the conviction that the human being can intervene in natural determinism, albeit completing or modifying it, by means of the manipulation of certain powers, accessible through aptitudes, knowledge, or special techniques” and superstition as “a knowledge or belief considered erroneous and not accepted by whoever has the authority to distinguish between legitimate and illegitimate knowledge” [91,92].

Superstition in medicine has been evaluated by Luis Gil in his work *Therapeia. La medicina popular en el mundo clásico *[93] in which he interprets the curative procedures of the animal-based medicine throughout history, with some words from MacKinney [94].

“It cannot be denied that the later Greek writings in medicine contain remedies that are more primitive than those of Hippocrates’ time. The same phenomenon, rise in superstitious prescriptions when a civilization ages, can be observed in other historical periods, especially in Ancient Egypt and Medieval Europe. However, this fact does not justify the conclusion that medical science gives way to superstition when a civilization ages. Thus, the writings of the later centuries contain an ever-increasing load of data, not only of science, but also of superstition, which includes that transmitted in previous eras. The Ebers Papyrus contains recipes from previous periods. The same is true of *De Materia Medica*, compiled by Dioscorides in the late period of the Greek civilisation” [93].

In zootherapy it can be affirmed that belief systems have been significant throughout history. Empedocles and Plato considered the importance of therapy not based on reason [95,96].

The magical theme of medicine is observed in the remedies compiled, in such a way that some are based on “transferential therapy”, where the illness or pain is redirected to the animal, as happens in the case of poisonous bites or stings. We can cite, for example, species that act as antidotes when applied directly to the skin, such as *Sarda sarda*, *Thunnus thynnus*, *Mullus* sp. (*M. barbatus barbatus*, *M. surmuletus*), *Trachinus draco*, *T. radiatus*, *Scorpaena porcus* or *Dasyatis pastinaca* (see Table 3).

Astrology exercised an influence on ancient pharmacopoeia by relating therapeutic capacity to favourable astral constellations. In the Zodiac Man or *homo signorum* of Burriana (province of Castellón) we can observe that a link was established between the astrological signs and various parts of the body [97]. Thus, we find that by presenting a woman in labour with a “tembladera” (*Torpedo marmorata*, *T. torpedo*), caught when the moon is in Libra, the delivery will be made easier. The astrological systems used animal symbols to explain how different parts of the human body are related to illnesses. These human body-animal associations seem to have largely originated from sympathetic magic, whereby each animal possesses specific facilities which can justify their relation to a certain part of the body, to which their “virtue” would be transmitted. In the Old World, fish, recognised for the agility of their movements, were related to feet [98]. Accordingly Table 3 shows folk remedies for the treatment of gout and knee and foot joints pains (*Anguilla anguilla*, *Lophius piscatorius*).

Dolores Carmen Morales has carried out an analysis of fish, noting the link between this animal group and reproductive apparatus and fertility:

“As for fish, the symbol of Jesus par excellence, they maintain their high reputation in Christianity due to their inheritance from other cultures. Psychic or sacred animals, fish have, however, been read in other ways: sexual interpretations because of their phallic form. Sacred Writings cite them on various occasions, possibly the most relevant reference being the miracle of the multiplying of the bread and fish. Fish are also a symbol of fertility because of the quantity of eggs produced” [99].

Thus, in Table 3 we see remedies for disorders or diseases related to the reproductive system, which follow a sympathetic magic where “like produces like”, as happens with the remedies that aid childbirth, based on species such as *Anguilla anguilla*, *Torpedo marmorata*, *T. torpedo*, *Silurus glanis* or *Dasyatis pastinaca*; increase the production of milk with *Spicara smaris* and *Gadus morhua*; and are used as aphrodisiacs, as *Anguilla anguilla* and *Pagellus erythinus*.

**Figure 4:**
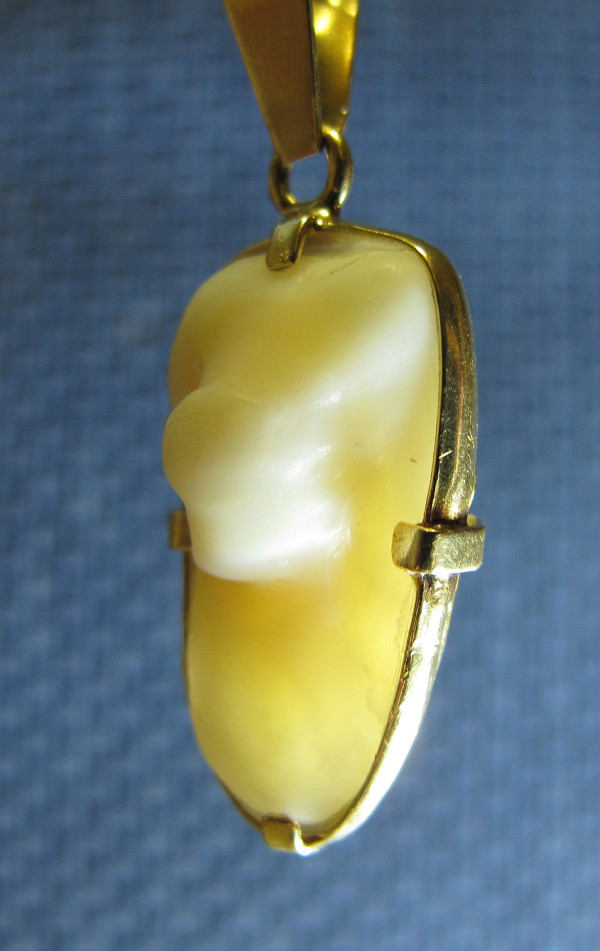
**“Piedra de corvina” (meagre fish white stone).** The otoliths of this fish species acquire a shape in which many people say that they see the face of the Virgin Mary, a fact that makes these mineral structures in a reputed protective amulet. In the pendant presented here we can see one of these otoliths mounted in gold. It belongs to the mother of one of the authors (JRV, Badajoz), who carries this jewel around her neck to prevent attacks of lumbago.

We find remedies based on sympathy associated to other systems, like the application of *Torpedo* sp. for the treatment of diseases related to the spleen, due to its similarity in form. In the same way, otoliths or “stones from some fish” (sensitive structures to gravity and linear acceleration) are associated with the treatment of kidney stones, as in *Argyrosomus regius* and *Merlangius merlangus* (Figure 4). The red colour of *Mullus barbatus barbatus* or *M. surmuletus* means that these species can be used for the treatment of menstrual disorders, and the form of the adhesive disc of the head of *Remora remora* explains its use in easing the implantation of the embryo in the uterus, as suggested in vernacular names “chupón”, “chupona”, “ventosa” (sucker, plunger, suction pad), typical of the Cádiz and Huelva coast [100].

Hippocratic medicine was the natural philosophical foundation for therapy since antiquity up to the development of biomedicine in the 19th century. Humoralism explains the physiology of the body in terms of a balance between the four humours or fluids: black bile, yellow bile, phlegm and blood. The excess or deficiency of these humours, as a consequence of life style, would provoke diseases, disorders or conditions that would have to be corrected in order to recover health [101,102]. Juan Sorapán de Rieros, in his work *Medicina española, contenida en proverbios vulgares de nuestra lengua* (1616), associates one of the humours –phlegm– with fish:

“And to understand this, that of the four humours, which are in our body, one of them is called phlegm, whose nature is cold, and humid like water. It forms mainly in the stomach, and more so in winter (according to the doctrine of Hippocrates) and with food stuffs that are cold and wet, sticky and difficult to cook. Part of this phlegm stays in the stomach, and part of it passes to the liver, where in time of need, it is perfected by the body’s natural heat is converted into blood. There are two differences of phlegm, one that is natural, and another that is not natural. The natural one is white and tasteless. The unnatural, is sour, or is salty, or glassy. Natural phlegm and things similar in their qualities can easily turn into each other, according to the philosopher’s doctrine. Fish, in nature is cold and wet, like phlegm, and can change into it. And in this sense it is true to say that, all fish is phlegm” [103].

In Hippocratic medicine treatment by opposites or antipathy, *contraria contrariis curantur*, is an overriding principle which is based on primitive associations between opposites [93]. Although it’s risky to interpret the remedies, we can glimpse the pharmacological conceptualization of this principle to counteract the heat and dryness of many symptoms that would be caused by stomach bile, for example by infectious diseases such as rabies, mumps, malaria and tetanus, taking advantage of the phlegm provided by *Hippocampus* sp., *Trachurus trachurus*, *Perca fluviatilis* or *Lophius piscatorius.* Digestive diseases that caused dryness or black bile would also be balanced with phlegm, thus *Coris julis*, *Spicara smaris*, *Sardina pilchardus* or *Silurus glanis* were used as laxatives.

It should be noted that although the Hippocratic Corpus constantly reported apathetic and allopathic treatments, you can also find homeopathic treatments within its pages [96,102]. Therefore we can suggest a Hippocratic origin for the remedies which use phlegm against phlegm, such as the treatment of respiratory problems with *Silurus glanis*.

The empirical trend based on discerning the type of remedy that was useful in the treatment of a given disease appears in the *Historia de la Farmacia* by Quintin Chirlone and Carlos Mallaina in 1865, where it states the following:

Is it not known, for example, that people with a putrid fever ask for acids and certain fish please those with leucorrhoea and that dysentery is characterized by a particular appetite for grapes? [104].

Thus, we can note that throughout history species have been selected and have been used in all historical periods because of their empirical effectiveness. Although looking back we can find critics of the popular empirical medicine such as Abü Muhammad 'Abd Allah b. Rusd in his work *Acerca del método de la técnica artesanal en la terapéutica*, in which he writes:

“It is clear that when a ‘doctor’ treats a patient with any substance, he thus provides Nature with a beginning and an end of order, whether it be in the genre of disease or of health. But if the ‘doctor’ is ignorant of this natural order and proper purpose and gives Nature any random beginning –I mean treating the sick with whatsoever– he may in essence be wrong but accidentally gets it right, causing more deaths than health. It is clear that ignorance of this method is the cause (fol. 79 r.) or at least one of the causes that the wise ascribes to the origin of sensation and the sensible. But we are talking about health and about disease, because most deaths occur due to the medicine” [105].

Despite the existence of detractive positions against zootherapy there currently exists objective data which demonstrate the efficiency and potential of fish as a therapeutic resource. In this regard, we underline the synthesis by Fariña Pestano *et al*. [6] which refers to the narcotic and analgesic active ingredients found in Tetraodontidae fish [106] –used in Japan with patients diagnosed with neurogenic leprosy and cancer [107]– to a cardiac stimulant obtained from *Eptatretus stoutii* Linnaeus, 1758, antitumor agents in *Dasyatis sabina* (Le Sueur, 1824), analgesics in *Taricha* sp.; well as to the properties of omega-3 fatty acids obtained from shark liver in preventing atherosclerosis, hypertension, in wound healing and the stimulation of the immune system of alcoxiglycerol [108] collected by Costa-Neto [109].

Recently, researchers at the University of Almería have undertaken a study of 12 commonly consumed fish species in the southeast of Spain, demonstrating that the liver of these fish is an excellent source of fatty acids of the omega-3 family [110]. These long chain polyunsaturated fatty acids are currently used in the treatment of degenerative diseases of the nervous system and in mental health [111].

As already mentioned, the historical and therapeutic value of the fish fauna in Spanish ethnomedicine is expressed in the survival of medicinal resources belonging to, at least seven species throughout history: *Acipenser sturio*, *Anguilla anguilla*, *Engraulis encrasicolus*, *Halobatrachus didactylus*, *Hippocampus* sp., *Scyliorhinus canicula* and *Squatina* sp. In addition, there are 10 species of which we have only obtained records from the 20th century: *Argyrosomus regius*, *Clupea harengus harengus*, *Cyprinus carpio*, *Dicologoglossa cuneata*, *Gadus morhua*, *Luciobarbus sclateri*, *Merluccius merluccius*, *Oxynotus centrina*, *Salmo trutta* and *Sardina pilchardus*. This is particularly significant, for it indicates a dynamic Spanish ethnomedicine capable of generating new therapeutic resources in recent times. For these reasons, and taking into account that ethnomedicine in Spain is a medical system that has been virtually totally replaced by biomedicine, there is a need for further research in line with the methods and goals of the Spanish Inventory of Traditional Knowledge [112].

Coinciding with Quave and Pieroni [113] we think it is necessary to validate ethno-pharmaceutically those animal remedies that have stood the test of time, for it is this survival that is proof of its therapeutic potential and its possible applications in the pharmaceutical industry. During the 20th century, the most important groups of pathologies treated with fish-based remedies are: infectious and parasitic diseases, followed by those related to pregnancy, childbirth and postpartum, and diseases of the musculoskeletal system and connective tissue (see Table 4).

**Table 4:** Groups of diseases treated by fish products in Spanish ethnomedicine

**Chapter**	**Title**	**C**_**i**_	**IC**_**i**_
			
I	Certain infectious and parasitic diseases	5	1.00
II	Neoplasms	1	0.20
III	Diseases of the blood and blood-forming organs and certain disorders involving the immune mechanism	1	0.20
IV	Endocrine, nutritional and metabolic diseases	2	0.40
V	Mental and behavioural disorders	3	0.60
VI	Diseases of the nervous system	—	—
VII	Diseases of the eye and adnexa	1	0.20
VIII	Diseases of the ear and mastoid process	—	—
IX	Diseases of the circulatory system	3	0.60
X	Diseases of the respiratory system	2	0.40
XI	Diseases of the digestive system	2	0.40
XII	Diseases of the skin and subcutaneous tissue	1	0.20
XIII	Diseases of the musculoskeletal system and connective tissue	4	0.80
XIV	Diseases of the genitourinary system	1	0.20
XV	Pregnancy, childbirth and the puerperium	4	0.80
XVI	Certain conditions originating in the perinatal period	2	0.40
XVII	Congenital malformations, deformations and chromosomal abnormalities	—	—
XVIII	Symptoms, signs and abnormal clinical and laboratory findings, not elsewhere classified	2	0.40
XIX	Injury, poisoning and certain other consequences of external causes	3	0.60
XX	External causes of morbidity and mortality	1	0.20
XXI	Factors influencing health status and contact with health services	1	0.20
XXII	Codes for special purposes	—	—

Significantly, those chapters of ICD-10 which are not represented are diseases of the nervous system and the ear and mastoid process, as well as congenital malformations, deformations and chromosomal abnormalities. Two out of three remedies used in the 20th century (74%) are empirical, based on the humorism and the principle of *contraria contrariis curantur* (“the opposite is cured with the opposite”). The rest, 26%, are magical type remedies that complete the popular therapeutic arsenal.

### A cross-cultural comparison

Although less important than other groups of vertebrates, fishes are represented in ethnopharmacological studies related to traditional medicine of different geographic regions and human communities [10,114-116]. This fact facilitates us to carry out a cross-cultural approach in relation to current fish-based zootherapies.

The medicinal species collected in this study are not used in remote areas of the northern hemisphere, but if they are employed species belonging to some documented genera. In India the eel (*Anguilla bengalensis* in this case) is used: its body mucus is applied on burned zones of the body [117], and fat is applied and massage to relieve rheumatoid-arthritis pain [118]. In the same way, at Jeju Island (Korea) the salted heads of *Engraulis japonica* are used to treat head lacerations [119].

Comparing with the data documented by other authors for other areas of the Mediterranean Region [113,120], firstly point out that the 17 fish species used in contemporary Spanish ethnomedicine constitute a very high number of therapeutic resources. And as to common species, the use of “bacalhau” (*Gadus morhua*) in Portugal is remarkable. In our neighboring country, this species is used against diseases such as anorexia, anemia and madness, in the treatment of abdominal pains and bone fractures, just like anti-fever and anti-parasitic [120].

These latter data induce us to discuss if there are species or similar (same family, for example) that also are used in the American countries were colonized by Spain and Portugal.

In Latin America the use of fauna with medicinal properties is a common practice since pre-Hispanic times. According to Foster [121] medical ideas and practices of indigenous peoples, along with the specific to the Spanish folklore and medieval and classical formulations have built a solid traditional medicine in this region of the world. Logically, the biogeographical differences between the Iberian Peninsula and the countries under colonization prevented the dissemination of a knowledge relate to the medicinal use of fishes at the species level. However, taxa belonging to families such as Carangidae, Engraulidae, Gadidae, Scombridae or Syngnathidae are/were used in both areas [122].

Brazil is the country where a greater number of species are used as medicines (85): 44 fresh-water species and 41 salt-water species [19]. According to a recent review [123], 77 species are consumed as food medicines. Among the fishes there documented the consumption of two genera coincides: *Dasyatis*, used in the Spanish medicine until the 17th century, and *Gadus*. In the latter case, *G. morhua* is the only species consumed, particularly in the treatment of furuncles. This species is also used in Brazil as a topical remedy for rheumatism and backache [19,122]. It can ensure that the Atlantic cod is a medicinal species used in force in Brazil, Portugal and Spain.

There is no doubt that the use of fishes, as a “general ethnotaxa”, must be part of a cultural transfer between the New and Old World as a consequence of symbolic nature [98]. Such cultural transfer stimulated the use of certain fish species similar to those in Europe, as well as their derivatives. For example, Francisco Hernández de Toledo (1514–1587) in his book *Cuatro libros de la naturaleza y virtudes de las plantas y animales de uso medicinal en la Nueva España* (published in Mexico in 1615) describes the use of otoliths against kidney stones [124], also used for the same purpose in Spain.

In the Argentine Gran Chaco region the venomous sting of the stingray *Potamotrygon* is used to eliminate the toothache [125] similarly to how the sting *Dasyatis pastinaca* was used until the early 17th century in Spain [36].

Likewise, in both geographical areas they are/were used dried seahorses (genus *Hippocampus*). In Spain, until the mid 20th century, these charismatic animals were only carried in the pocket or round the neck as amulets to prevent erysipleas and cure toothache (see Table 3). No longer are roasted and eaten. Conversely, in Brazil the longsnout seahorse (*Hippocampus reidi*) is among the most versatile fish species in terms of therapeutic indications [19,126]. This species is still consumed by its intake as part of different mixtures (teas, concoctions, etc.) with various medicinal plants and animals. These medicinal and/or superstitious uses put these fishes among the most traded animals for medicinal purposes. This led to the entire genus was included in May 2004 in the Appendix II of CITES, to ensure that trade is not detrimental to the survival wild populations [126].

## Conclusions

Historically, fish-based therapeutics is based on a dietary usage; it has been an essential product in the care and maintenance of health from antiquity to the present. However, throughout history a wide range of pathologies have been treated through the use of these animals, and some remedies have survived to this day, highlighting especially those related to infectious and parasitic diseases, pregnancy, childbirth, puerperium and diseases of the musculoskeletal system. There is also a cultural merging, or syncretism detected in the remedies and a strong relationship exists between magic and empiricism. In the last century both were almost equally present, a period in which we also find a progressive decrease in the number of fish species used. Seven species have been documented as surviving therapeutic resources since centuries ago; the existence of a dynamic Spanish ethnomedicine has also been detected which has managed to generate new therapeutic resources in recent times. Historical ethnozoology can be a transverse axis in the history and philosophy of science; it may participate in the establishment of cultural parallels and even as ethnopharmacological research support. Despite the limited interest shown in zootherapy in Spain, it is important to evaluate it along with the rest of traditional ecological knowledge, much of which has been validated by ethnopharmacology and evidence-based medicine. In order to recover as much data as possible, it will be necessary to draw up an inventory of ethnoichthyological uses, so that it could act as an anamnesis of remedies together with the data compiled in this study. It is also important to determine the cultural significance of fish-based zootherapy and to further watch and determine how globalization and multiculturalism are influencing.

## Consent

Informed consent was obtained from the people who appear in the photograph for the publication of the accompanying image.

## Competing interests

Both authors declare that they have no competing interests.

## Authors’ contributions

The two authors contributed equally during the data collection, data analysis and preparation of the manuscript, and read and approved the final manuscript.
